# Relation extraction with weakly supervised learning based on process-structure-property-performance reciprocity

**DOI:** 10.1080/14686996.2018.1500852

**Published:** 2018-09-19

**Authors:** Takeshi Onishi, Takuya Kadohira, Ikumu Watanabe

**Affiliations:** a Toyota Technological Institute at Chicago, Chicago, IL, USA; b Research and Services Division of Materials Data and Integrated System, National Institute for Materials Science, Tsukuba, Ibaraki, Japan; c Research Center for Structural Materials, National Institute for Materials Science, Ibaraki, Tsukuba, Japan

**Keywords:** Natural language processing, knowledge extraction, relation extraction, weakly supervised learning, materials informatics, 60 New topics/Others, 404 Materials informatics / Genomics

## Abstract

In this study, we develop a computer-aided material design system to represent and extract knowledge related to material design from natural language texts. A machine learning model is trained on a text corpus weakly labeled by minimal annotated relationship data (~100 labeled relationships) to extract knowledge from scientific articles. The knowledge is represented by relationships between scientific concepts, such as {*annealing, grain size, strength*}. The extracted relationships are represented as a knowledge graph formatted according to design charts, inspired by the process-structure-property-performance (PSPP) reciprocity. The design chart provides an intuitive effect of processes on properties and prospective processes to achieve the certain desired properties. Our system semantically searches the scientific literature and provides knowledge in the form of a design chart, and we hope it contributes more efficient developments of new materials.

## Graphical Abstract

**Figure:**
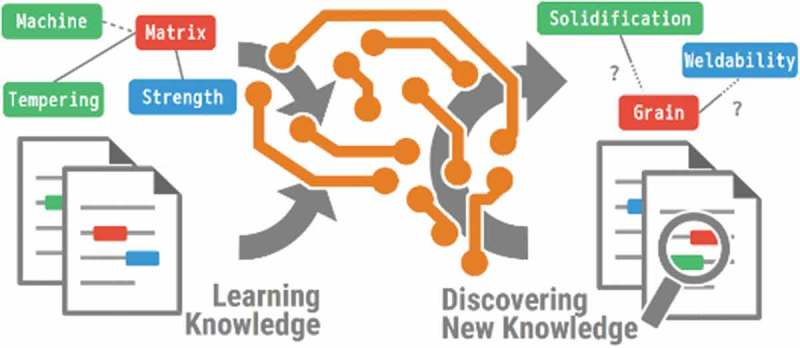

## 1. Introduction

Machine learning and data science for knowledge extraction are studied in a wide variety of field. Knowledge extraction is to find desired knowledge from text. For example, relationships among scientific knowledge are extracted from scientific literature in ScienceIE [1], and a knowledge base is extracted from Web text in TAC (https://tac.nist.gov). The impressive performance of machine learning appears promising for knowledge extraction in material design as well.

Material design is a process of developing new materials with specific properties. In most practical cases, the desired process cannot be envisioned, and instead it is constructed by trial and error. In this approach, a trial is a time-consuming experiment. Minimizing the number of trials is critical for an efficient development. On the contrary, such efficient development is challenging because 1) The relation between a process and a property is unclear and indirect; and 2) The search space (the set of possible processes) is too large to look up.

In practice, researchers find these processes relying on their end-to-end knowledge including effects of processes to the properties. Such knowledge is technical and might not be well formalized, so they spend long time to obtain such knowledge. We believe it is beneficial to provide the end-to-end knowledge for accelerating material developments.

### 1.1. Knowledge representation

The processing-structure-property-performance (PSPP) reciprocity [2] explains effect of processes on properties in three stages. The first stage is ‘process’ that can be controlled to develop a new material. The second stage is ‘structure’ of the material that the processes build. The third stage is ‘property’ that the structure gives. The properties in the third stage give the total performance of the new material.

**Figure 1.**
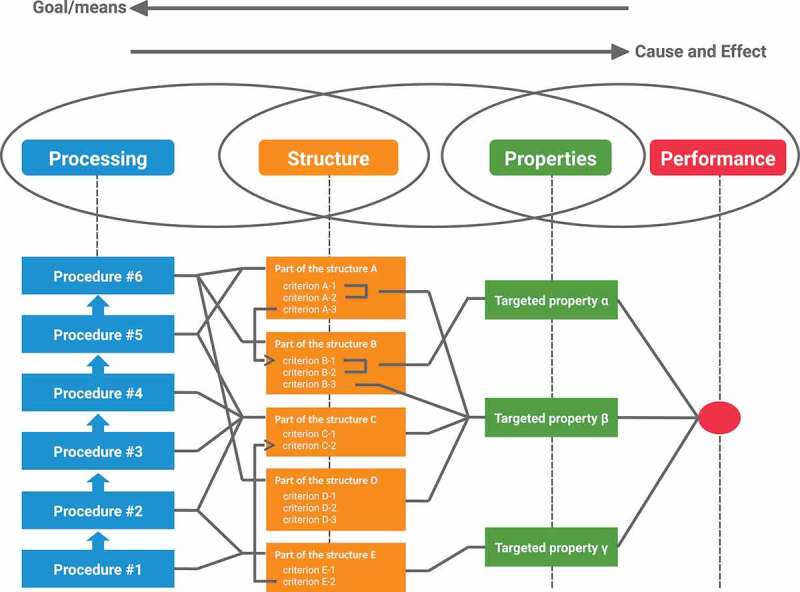
The process-structure-property-performance reciprocity.

The PSPP design chart [3] represents end-to-end knowledge in the form of relationships among factors, as shown in Figure 1 [4]. A factor represented by a node is an important phenomenon or concept for material design, such as *annealing, grain size*, and *strength*. A factor is classified into one of the three stages, ‘process’, ‘structure’, and ‘property’, where the factor performs. For example, *annealing* is an important concept in ‘process’. Following the PSPP reciprocity, a factor is influenced by connected factors to its left, and influences factors connected to its right (i.e. a process builds structures, and a structure influences properties of the material). These relations are represented by their connectivities. The chart intuitively represents end-to-end knowledge in the form of relationships between processes and properties mediated by structures [5].

### 1.2. Data limitation

Despite major developments in machine learning, the technology suffers from limited data availability for model training in practical cases. For example, AtomWork [6], one of the largest databases available for material design, contains records of more than 55,000 properties of materials. However, the knowledge in the database unlikely covers all knowledge needed for material design. For example, AtomWorks covers crystal structures and related properties such as lattice constant and space group but microstructures of materials are unlisted. Such limited data likely lead to over-fitting during training, and thus to poor performance.

In this study, we aim to overcome the problem of limited data availability using Natural Language Processing (NLP), an application of machine learning technologies to natural language resources, such as scientific articles and Web texts. Natural language resources are widely available and are machine readable, for a variety of fields including material design. For example, Elsevier’s API (https://dev.elsevier.com) provides access to over 250,000 fully digitized scientific articles. More importantly, natural language is the most popular means of representing knowledge, and our desired knowledge is thus likely to be present.

### 1.3. Weakly supervised learning

We leverage weakly supervised learning to identify relationships in the PSPP chart. Weakly supervised learning is used to train a model with a minimal number of annotations for relation identification [7]. In a typical supervised setting, the training data for relation identification is a sentence labeled with entities in the sentence and the relations among them. However, labeling sentences is expensive because an annotator must read a sentence, and label the entities and relations described therein. This renders each label clean and strong. On the contrary, in a weakly supervised setting, a knowledge base produces a pair of entities and their relations and all sentences containing these entities are weakly labeled with the given relation. For instance, for the given entities, **Elevation Partners** and **Roger McNamee**, and their relation *founded*_*by*, both of the following sentences are labeled with *founded*_*by*,

**Elevation Partners**, the $1.9 billion private equity group that was founded by **Roger McNamee;**

**Roger McNamee**, a managing director at **Elevation Partners**,…;

where the first sentence describes the relation *founded_by* but the second sentence does not. Weak labels do not require any annotations but the knowledge base, however; they are noisy, and a model needs to overcome the noisy labels.

In recent years, convolutional neural network (CNN) models have surpassed feature-based models [7–11]. CNNs are a class of neural networks with convoluted neural units. Residual learning is used to help the deep CNN network [12]. Zeng et al. [13] split a sentence into three parts, and then applied max pooling to each part of the sentence over a CNN layer.

Sentence-level attention is introduced for selecting a key sentence. In this approach, a network takes a set of sentences for a relation between two entities. Each sentence contains both entities. An attention mechanism over a CNN allows the network to automatically select a key sentence, which is likely describing the desired relation. It seems helpful to overcome the problem of noisy labels [14–16].

### 1.4. Our contribution

We develop a computer-aided material design (CAMaD) system with the aim of generating a PSPP design chart for desired properties from text. The PSPP chart suggests prospective processes to achieve given arbitrarily desired properties. The system is based on a machine learning model for relation extraction from text. The model is efficiently trained with weakly supervised learning, which minimizes the annotation cost of the training data. We believe the PSPP chart helps more efficient material developments by suggesting a prospective process.

Our contribution in this study is twofold. First, we proposed a novel knowledge graph based on PSPP charts and developed a system to build the knowledge graph from text using NLP technologies. Second, we experimentally verified that such technical knowledge can be extracted from text using machine learning models. Our target knowledge is relations in PSPP design charts. These relations appear rather technical and significantly different from typical relations in NLP such as ‘has_a’ and ‘is_a’. Extraction of these relations from text only is difficult and might need other knowledge resource such as equations and properties of materials. We, however, experimentally verified that a state-of-the-art machine learning model can extract these relations from text.

In the following sections, we formalize our task with three subtasks in Section 2 and describe our pipeline system for each subtask in Section 3. We are especially interested in the second subtask; we evaluated our system for the subtask in Section 4, and present the results in Section 5. We also briefly describe the end-to-end implementation in Section 6, and future works in Section 7.

## 2. Our approach and task definition

Our approach is knowledge graph population and graph search. The knowledge graph represents knowledge concerning material design. It consists of factors and their relations following PSPP reciprocity, and a PSPP chart is considered as a part of the graph with factors related to desired properties. Thus in this approach, we first extract the structure of the graph from text (knowledge graph population) and then find a PSPP chart from the graph for a desired material (graph search).

The PSPP knowledge graph consists of nodes and edges between them. Each node represents a factor, an important concept in material design. A factor is classified into one of ‘process’, ‘structure’, and ‘property’. Each edge is a relation between factors represented by the nodes. The nodes are connected by the edge if and only if the corresponding factors are related in the PSPP chart. Therefore, there is no edge between processing factors and structural factors.

A PSPP chart is considered part of the PSPP knowledge graph for a developing material. For instance, considering a typical material development scenario, where a new material is desired with specific properties, the PSPP chart is composed of factors related to the desired properties. The PSPP chart is part of the PSPP knowledge graph around the nodes of the desired property factors.

Following this approach, the task is decomposed into three subtasks: factor collection, relation identification, and branching.

The first subtask is to collect factors for nodes in the PSPP knowledge graph, and these factors are classified into each PSPP class. A factor is an important scientific concept for material design and is classified into ‘process’, ‘structure’, or ‘property’ following PSPP reciprocity. For example, process factors include ‘tempering’ and ‘hot working’; structural factors include ‘grain refining’ and ‘austenite dispersion’; and property factors include ‘strength’ and ‘cost’. Each factor is represented by a node in the PSPP knowledge graph, i.e. a node represents a processing, structural, or property factor.

The second subtask is relation identification, where relations among nodes in the PSPP knowledge graph are identified by reading text. In this subtask, for two given nodes and sentences mentioning the factor represented by these nodes, their relation is identified. Following PSPP charts, the relation is labeled in binary manner, i.e. positive or negative. A positive relation between factors A and B indicates that ‘*factor A affects factor B*’, and a negative relation between factors A and C indicates that ‘*factor A occurs independently of factor C*’. In a chart, two nodes are connected if their factors of the nodes have a positive relation, and are otherwise unconnected. Thus, denoting a pair of factors by $\left(f_{1},f_{2}\right)$, the desired relation is

(1) $$h(f_{1},f_{2})=pos/neg.$$

The third subtask is to obtain a PSPP chart by branching the PSPP knowledge graph. We assume a scenario where a scientist is developing a new material with certain desired properties and looking for factors related to the properties in a PSPP chart. In this scenario, the PSPP chart is part of the PSPP knowledge graph, with certain factors around the desired properties. Thus, the subtask is to find part of the PSPP knowledge graph given a set of properties.

## 3. System description

Our system is a pipeline system consisting of three components for each subtask. The first component collects factors from a keyword list (Section 3.1). In the second component, a relation between two nodes is identified by reading sentences containing the factors represented by the nodes in the CNN model (Section 3.2). In the third component, part of the PSPP knowledge graph is extracted for the given desired properties by a simplified maximum flow algorithm (Section 3.3).

### 3.1. Factor collection

First, factors are collected from the keyword list of the journal Scripta Materialia (https://www.journals.elsevier.com/scripta-materialia). Such keywords help identify the topics of each article. The keyword list is divided into five sections: 1) Synthesis and Processing; 2) Characterization; 3) Material Type; 4) Properties and Phenomena; and 5) Theory, Computer Simulations, and Modeling. In this approach, keywords in synthesis and processing are collected as processing factors, those in material type are collected as structural factors, and keywords in properties and phenomena as property factors.

**Figure 2.**
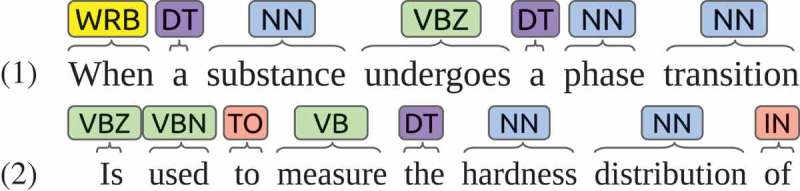
Sentences containing noun phrases.

Second, structural factors are collected from text using linguistic rules. From a material science standpoint, the number of structural factors is significantly greater than those of processing and property factors, and the keyword list is not long enough to cover structural factors. Candidate phrases, noun phrases consisting of multiple NNs (singular nouns, or mass nouns), are collected from a corpus described in Section 4.2 using Stanford CoreNLP [17]. Each candidate phrase is classified into structural factors if it does not contain any words in the keyword list. The phrase containing a keyword is classified as the class of the keyword. For instance, Figure 2 lists two sentences with noun phrases. Here ‘phrase_transition’ is classified as a structural factor, but ‘hardness_distribution’ is classified as a property factor, as ‘hardness’ is in the keyword list.

All keywords and the n most frequent candidate phrases are collected, and each word/phrase is assigned a node in the PSPP knowledge graph. The total number of factors was 500, 500, and 1000 for process, property, and structural factors, respectively. Table 1 lists samples of the n most frequent phrases.

**Table 1.** Samples of factors obtained by the linguistic rules.

Process	Structure	Property
water quenching	carbon dioxide	creep behavior
element modeling	grain distribution	fatigue behavior
peak temperature	particle size distribution	misorientation angle
rolling texture	matrix phase	shock resistance
deformation mode	β titanium alloy	fracture strain
microwave sintering	β grain size	tensile ductility
plasma sintering	solution strength	fracture behavior
discharge machining	pore size	vacuum induction melting

### 3.2. Relation identification

In this section, we describe our CNN model for identifying the relation of a factor pair by weakly supervised learning. We also describe the linguistic resource for the factor pair where the model was trained.

The linguistic resource of a factor pair is a set of sentences mentioning both factors. A mention is a part of a sentence referring to a factor. A factor is mapped to the mention in the sentence by max-span string matching, i.e. a factor is mapped to the mention if the mention is the factor name, and no other mention overlaps the given mention. For instance,

• Within each **phase**, the properties are …

• When a substance undergoes a **phase** transition …

The **phase** in the first sentence is mapped to a factor, ‘phase’, but **phrase transition** is mapped to ‘phase_transition’ instead of ‘phase’ in the second sentence. A sentence in the linguistic resource of a factor pair is a sentence mentioning both factors.

**Figure 3.**
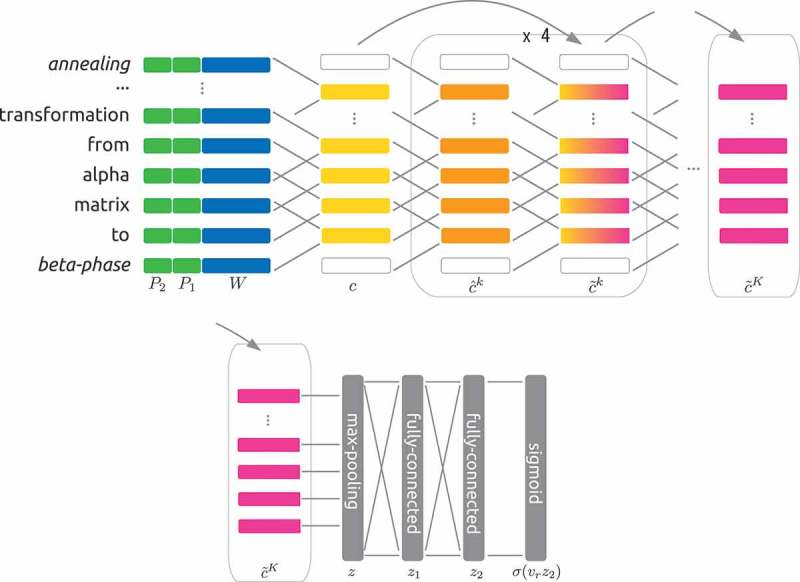
Structure of the CNN model. The convolutional layers embed a sentence, and the max pooling and two fully connected layers give a binary probability distribution with a sigmoid function.

The CNN model proposed by Huang et al. [12] is a state-of-the-art deep neural network model for weakly supervised relation extraction. For each sentence in a linguistic resource, the network takes word embeddings and the relative position embeddings toward factors in the sentence. Convolutional units with a deep residual learning framework then embed the sentence into a vector representation. The sigmoid layer over the vector representation produces the probability distribution of the binary relation. Figure 3 shows the overall structure of the network.

The input to the model is a sentence and the output is a relation $r\in \left\{pos,neg\right\}$. Let $\left(f_{1},f_{2}\right)$ be factors of the relation and $s\in S_{f_{1},f_{2}}$ be the sentence, i.e. the linguistic resource, $S_{f_{1},f_{2}}$ is a set of sentences mentioning the factors. Each sentence s is padded to a fixed length L.

A token embedding is a vector representation of a token in a sentence, denoting the sentence $s=\left\{t_{0},...,t_{i},...\right\}$, where $t_{i}$ is the ith token. The token representation is $x_{i}$, which is a concatenation of a word embedding and two position embeddings. A word embedding W(⋅) is a vector representation of the word of the token whereas a position embedding P(⋅) gives a vector representation of the relative position of each factor:

(2) $$x_{i}=[W(t_{i});P_{1}(k_{1}-i);P_{2}(k_{2}-i)]$$

where $W(\cdot )\in R^{d_{w}}$, $P(\cdot )\in R^{d_{p}}$ and $k_{1},k_{2}$ are the position indices of each factor. Note that any relative distance greater than $D_{max}$ is treated as $D_{max}$.

A convolution layer takes embeddings around position i, and maps them into $c_{i}\in R^{d_{c}}$:

(3) $$c_{i}=g(wx_{i:i+h}+b)$$

where $x_{i:i+h}=[x_{i};x_{i+1};...;x_{i+h-1}]$, $w\in R^{d_{c}\times h(d_{w}+2d_{p})}$ and $b\in R^{d_{c}}$ is a bias. g is an element-wise non-linear function, ReLU.

Following the first convolutional layer, the other layers are stacked with residual learning connections that directly transmit a signal from a lower to a higher layer while skipping the middle layers. Thus, the kth residual CNN block consists of two CNN layers, with one taking signals from the two lower layers:

(4) $${\hat{c}}_{i}^{k}=g({\hat{w}}_{k}({\tilde{c}}_{i:i+h}^{k-1}+{\tilde{c}}_{i:i+h}^{k-2})+{\hat{b}}_{k})$$

(5) $${\tilde{c}}_{i}^{k}=\;\;\;\;g({\tilde{w}}_{k}{\hat{c}}_{i:i+h}^{k}+{\tilde{b}}_{k})$$

where ${\tilde{c}}^{0}=c$. The first CNN layer ${\hat{c}}_{i}^{k}$ takes a signal from the immediately lower layer ${\tilde{c}}_{i:i+h}^{k-1}$ and another signal from the lower block ${\tilde{c}}_{i:i+h}^{k-2}$.

Max pooling is performed over the output of the last CNN units, ${\tilde{c}}^{K}\in R^{L-h+1\times d_{c}}$.

(6) $$z=\underset{i}{maxpool}\;{\tilde{c}}_{i}^{K}$$

Then, two fully connected layers and a sigmoid function yield the probability distribution of the desired relation given in the sentence P(r=pos/neg|s):

(7) $$z_{1}=g(w^{g_{1}}z+b^{g_{1}})$$

(8) $$z_{2}=g(w^{g_{2}}z_{1}+b^{g_{2}})$$

(9) $$P(r=pos|s)=\sigma (v_{r}z_{2})$$

where $w^{g}\in R^{d_{c}\times d_{c}}$ and $b^{g}\in R^{d_{c}}$.

The desired probability $P(r=pos|\;f_{1},f_{2})$ is the maximum of the probabilities over sentences. This is

(10) $$P(r=pos|\;f_{1},f_{2})=\underset{s\in S_{f_{1},f_{2}}}{\max}P(r=pos|s)$$

On the contrary, the model is trained on a weakly supervised approach, where the objective function is maximized for each sentence.

(11) $$\underset{\Phi }{max}\underset{(f_{1},f_{2},r)\in D_{train}}{\sum }\underset{s\in S_{f_{1},f_{2}}}{\sum }\log P(r|s)$$

where $D_{train}$ is the training data, a set of tuples of factors $f_{1},f_{2}$ and relation $r\in \left\{pos,neg\right\}$. The parameters $\Phi =\left\{W,P_{1},P_{2},w,b\right\}$

### 3.3. Branching

The PSPP knowledge graph is branched for the given desired properties without losing related factors. We consider the branching of a max-flow problem, where the flow occurs from the given property factors to the processing factors. The inlets are all nodes of the processing factors and the outlets are those of the given properties. The capacity of each edge is the score of the relation, i.e. $P(r=pos|f_{1},f_{2})$. We maximize the amount of flow with a limited number of nodes in the graph.

We compute the capacity of a node in the graph, which is the amount of flow that it can accept. Recalling that nodes of structural factors are connected to property and processing factors, and no processing factor and property factor are connected, all flow pass through the nodes of the structural factors. The capacity of the node of a structural factor $f_{str}$ is

(12) $$C_{f_{str}}=min\left(\underset{f\in PRC}{\sum }P(r=pos|\;f,f_{str}),\underset{f\in {PRP}^{\prime }}{\sum }P(r=pos|\;f_{str},f)\right)$$

where PRC represents processing factors and PRP’ is the desired property factors. Similarly, the capacity of a node of a processing factor $f_{prc}$ is

(13) $$C_{f_{prc}}=\underset{f\in {STR}^{\prime }}{\sum }P(r=pos|\;f_{prc},f)$$

where STR’ represents structural factors that are not branched.

The desired PSPP chart is composed of n processing factors, m structural factors, and the desired property factors, where n and m are the given hyper-parameters. The nodes of the processing/structural factors are the n and m most capable nodes. For efficiency, the nodes are greedily searched such that optimality is not guaranteed. The PSPP chart shows the processing/structural factors related to the desired properties.

## 4. Experiment for relation identification

Relation identification is a challenging subtask in this research. The system performance on the subtask was evaluated in a weakly supervised relation extraction setting. In this evaluation, the relation was identified as positive/negative for each factor pair.

The training data consisted of relationship data and a corpus. The relationship data consisted of a pair of factors and its relation label. The corpus, scientific literature, was a set of sentences describing the factors. A model was trained on part of the relationship data and the corpus and was tested on held-out data and the corpus by predicting relationships in the held-out data.

Our model was trained using stochastic gradient descent and dropout. Dropout randomly drops some signals in the network that are thought to help the generalization capabilities of the network. We employed an Adam optimizer with a learning rate of 0.00005 and randomly dropped signals from max pooling during training with a probability of 20%. The word embeddings were initialized with GloVe vectors [18]. Other hyper-parameters are listed in Table 2.

**Table 2.** Hyper-parameters of the CNN model.

Parameter	Value
L	100
$D_{max}$	30
K	4
h	2
$d_{w}$	50
$d_{p}$	5
$d_{c}$	50
$L_{2}$ regularization	0.0001

### 4.1. Relationship data

The relationship data consisted of tuples of two factors and their binary relation (pos/neg). From four design charts [5], 104 factor pairs were collected as shown in Tables 3 and 4.

**Table 3.** Factors in the relationship data.

Category	Size
Process	17
Structure	21
Property	6

**Table 4.** Relations in the relationship data.

Relationship type	Positive	Negative
Process ↔ Structure	14	49
Structure ↔ Property	10	31

For evaluation, our model was trained on part of the relationship data and tested on the held-out data. The training data consisted of relationships from three arbitrary charts out of the four, and the test data consisted of relationships in the fourth chart. Thus, four pairs of training and test data were prepared for the evaluation. For accurate evaluation, the likelihoods of relationships in the test data were computed by a model trained using the corresponding training data. Precision and recall curves were then computed for the overall relationship data to obtain a smooth curve.

### 4.2. Corpus

Our corpus consisted of publicly accessible scientific articles on ScienceDirect (https://www.sciencedirect.com). ScienceDirect is an Elsevier platform providing access to articles in journals in a variety of fields, such as social sciences and engineering. Approximately 3400 articles were collected using the keyword search on ScienceDirect. The keywords were ‘material’ and ‘microstructure’, i.e. each article was related to both ‘material’ and ‘microstructure’.

The CNN models were trained on a pair of factors and sentences. As described in Section 3.2, each sentence mentions both factors. For the relationship data, about 5000 sentences were founded in the corpus in total, roughly 50 sentences for each pair of factors on average.

### 4.3. Baseline models

A baseline model is a text-classification-based binary classifier where for each factor pair, each classifier takes a set of sentences mentioning the factors and classifies the text into a positive or negative relation. The problem setting and the set of sentences were exactly the same as the one in the CNN model.

Logistic regression and SVM with bag-of-words features were employed for the binary classifier. These are standard machine learning binary classifiers. Bag-of-words is a feature that indicates whether a word is in a set of sentences. The feature is represented by a sparse binary vector, where an element is one if the corresponding word is in the sentences and zero otherwise.

**Figure 4.**
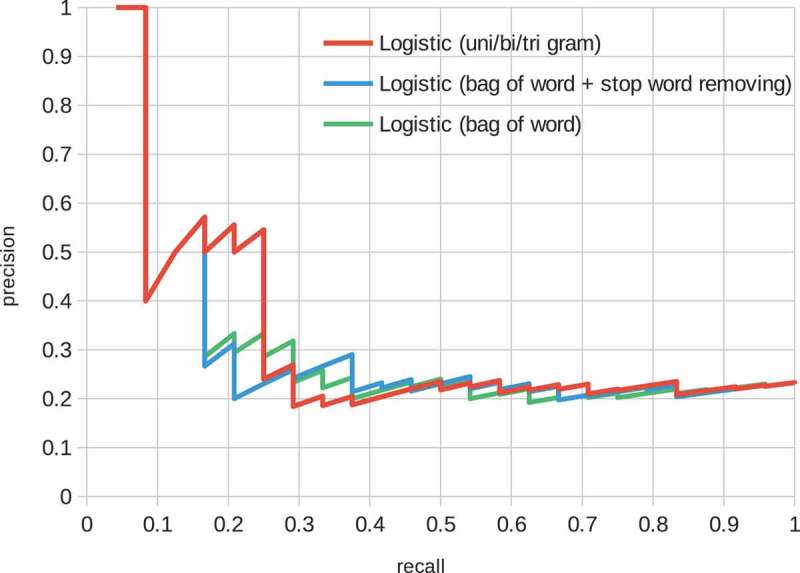
Precision-recall curve of the logistic regression model. The features are ‘bag of words’, ‘bag of words + stop word removal’ and ‘bag of unigram + bigram + trigram’.

**Figure 5.**
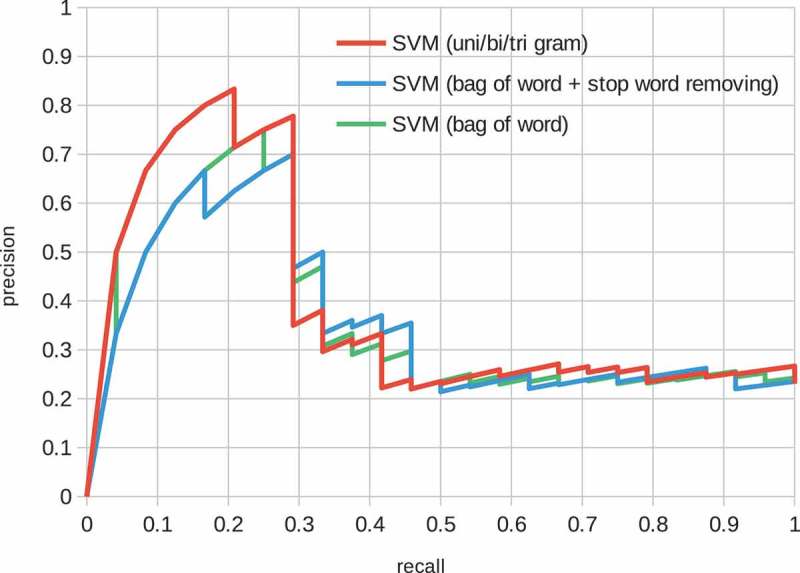
Precision-recall curve of the SVM model. The features are ‘bag of words’, ‘bag of words + stop word removal’ and ‘bag of unigram + bigram + trigram’.

Stop words removal and n-gram features are explored in Figures 4 and 5; however, the effect was limited. Note that the radial basis function (RBF) kernel was used in all SVM models.

### 4.4. Evaluation metric

The evaluation metrics were precision and recall, which are the standard metrics for information extraction tasks. Precision is the ratio of correctly predicted positive factor pairs to all predicted positive factor pairs and gives the accuracy of the prediction. Recall is the ratio of correct predictions to all positive factor pairs in the test data and gives the coverage of the prediction. A positive factor pair is a pair whose relation is positive. We obtain high precision and low recall if a system returns only a small number of high confidence predictions, and low precision and high recall if a system returns many low confidence predictions. Typically, these are balanced by a hyper-parameter (confidence) of system prediction. Thus, the trajectory of precision and recall pairs is computed with various values of the hyper-parameter and is called as a precision-recall curve.

In this evaluation, the hyper-parameter was an integer t, the number of positive factor pairs in the prediction. For a given t and a set of factor pairs in the test relationship data, the system predicts a binary relation, pos/neg, for each pair. It predicts the t most likely positive pairs, and the other pairs are predicted as negative.

The factor pairs in the test relationship data were scored by a machine learning model trained on the corresponding training relationship data, where the score was $P(r=pos|f_{1},f_{2})$. A test data corresponded with a training data, unaware of the relationships in the test data (Section 4.1). A model was trained on the corresponding training data and scored a pair in the test data to avoid letting the model know the true relationships during training.

Then, a precision and recall pair for a given hyper-parameter t was computed as follows:

(14) $$Precision_{t}=\frac{\left|R_{t}\cap R_{test}\right|}{t}$$

(15) $$Recall_{t}=\frac{\left|R_{t}\cap R_{test}\right|}{\left|R_{test}\right|}$$

where $R_{test}$ is the set of factor pairs with positive relations in all test relationship data, and $R_{t}$ represents the t most likely positive factor pairs. The likelihood was a score given by the model.

## 5. Results of relation identification

Precision-recall curves for the baseline models are shown in Figures 4 and 5. These figures show various feature representation schemes, such as stop words and n-grams (Section 4.3) on the logistic and SVM models. The logistic model performed well on low recall space, i.e. most confidently predicted positive factor pairs were actually positively related. On the contrary, the performance of the SVM model was poorer in the space but better overall than the logistic model. In both models, the effects of the feature representation schemes were limited.

**Figure 6.**
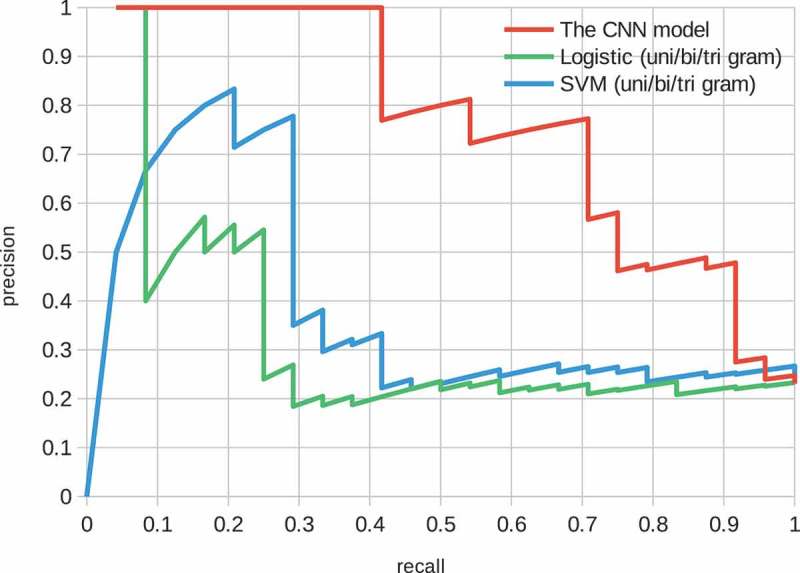
Precision-recall curve over the relationship data of the CNN model.

The performance of the CNN model is shown in Figure 6. The precision was one when the recall was about 0.4, i.e. roughly speaking half the positive factor pairs were perfectly identified. The performance was superior to that of the baseline models.

Table 5 shows some representative sentences scored by the CNN model. A representative sentence is the highest scored sentence in a sentence set $S_{f_{1},f_{2}}$ for each factor pair, i.e. a representative sentence is $s^{\prime }=argmax_{s\in S_{f_{1},f_{2}}}P(r=pos|s)$. The score is a likelihood where a positive relation is described in the sentence. The sentence with the highest score in each sentence set most likely represents the positive relation of each factor pair, according to the CNN model.

**Table 5.** Sample representative sentences scored by the CNN model. Label P indicates that the factors are positively related in the test relationship data and label N indicates a negative relation. Factors in each sentence are underlined. The score is the $v_{r}z_{2}$ of each sentence. See Appendix for the source articles.

	Score/Label	Sentence
1	36.5/P	… the following matrixform: [11] k∼u=λu …
2	34.8/P	… δc=rσc/τ is the characteristic or critical whisker length, f and r … τ is the matrix shear strength …
3	34.2/P	… toughness (δkcb) and grain … dvpwhere, d is the matrix …
4	31.0/P	… cast iron has a pearlite matrix and …
5	28.6/P	after solution treatment, the increase of grain size was not obvious because of the heat resistance introduced by … .2) after aging … .3) grain refining, size reduction of …
6	26.0/N	solution strengthening and precipitation strengthening respectively, …, δh−p was the yield strength …
7	24.7/N	…dislocation density in lath martensite matrix due to the high content of element … 100 steel delayed the recovery process during tempering …
8 . . .	23.8/P	lath martensite, which benefited the impact toughness …
9	−13.1/P	… the effect of ingot grain refinement on the mechanical properties of al profiles which are manufactured through hot working …
10	−14.1/N	… refining the prior austenitic grain size … LONG CONTEXT … the mechanical strength and cleavage resistance …
11	−16.4/N	… enhanced solid solution strengthening and composition homogenization is larger than …
12	−18.7/N	… as the solution treatment temperature increases to …, the transformation … and the formation of rim o phase …
13	−23.4/N	… during the aging treatment, the rim o phase at the margin of α2 grains become …

**Appendix.** Source articles.

1	B. Wen and N. Zabaras. Investigating variability of fatigue indicator parameters of two-phase nickel-based superalloy microstructures. DOI: https://doi.org/10.1016/j.commatsci.2011.07.055
2	L. Huang and Y. Chen. A study on the microstructures and mechanical properties of Ti-B20–0.1B alloys of direct rolling in the α+β phase region. DOI: https://doi.org/10.1016/j.jallcom.2015.05.244
3	Z. Yin, J. Yuan, Z. Wang, H. Hu, Y. Cheng and X. Hu. Preparation and properties of an Al2O3/Ti(C,N) micro-nano-composite ceramic tool material by microwave sintering. DOI: https://doi.org/10.1016/j.ceramint.2015.11.082
4	O. Oloyede, T. D. Bigg, R. F. Cochrane and A. M. Mullis. Microstructure evolution and mechanical properties of drop-tube processed, rapidly solidified grey cast iron. DOI: https://doi.org/10.1016/j.msea.2015.12.020
5	C. Shi, L. Zhang, G. Wu, X. Zhang, A. Chen and J. Tao. Effects of Sc addition on the microstructure and mechanical properties of cast Al-3Li-1.5Cu-0.15Zr alloy. DOI: https://doi.org/10.1016/j.msea.2016.10.063
6	C. Wang, C. Zhang, Z. Yang, J. Su and Y. Weng. Microstructure analysis and yield strength simulation in high Co-Ni secondary hardening steel. DOI: https://doi.org/10.1016/j.msea.2016.05.069
7	X. Shi, W. Zeng, Q. Zhao, W. Peng and C. Kang. Study on the microstructure and mechanical properties of Aermet 100 steel at the tempering temperature around 482°C. DOI: https://doi.org/10.1016/j.jallcom.2016.04.087
8	H. Xie, L.-X. Du, J. Hu, G.-S. Sun, H.-Y. Wu and R.D.K. Misra. Effect of thermo-mechanical cycling on the microstructure and toughness in the weld CGHAZ of a novel high strength low carbon steel. DOI: https://doi.org/10.1016/j.msea.2015.05.033
9	W. Haigen, X. Fuzhong and W. Mingpu. Effect of ingot grain refinement on the tensile properties of 2024 Al alloy sheets. DOI: https://doi.org/10.1016/j.msea.2016.11.016
10	A. Di Schino and C. Guarnaschelli. Effect of microstructure on cleavage resistance of high-strength quenched and tempered steels. DOI: https://doi.org/10.1016/j.matlet.2009.06.032
11	F.L. Cheng, T.J. Chen, Y.S. Qi, S.Q. Zhang and P. Yao. Effects of solution treatment on microstructure and mechanical properties of thixoformed Mg2Sip/AM60B composite. DOI: https://doi.org/10.1016/j.jallcom.2015.02.147
12, 13	X. Chen, F.Q. Xie, T.J. Ma, W.Y. Li and X.Q. Wu. Microstructural evolution and mechanical properties of linear friction welded Ti2AlNb joint during solution and aging treatment. DOI: https://doi.org/10.1016/j.msea.2016.05.030

Representative highly scoring sentences seem to describe the desired relations (sentences 4 and 8) and, interestingly, relations described in the equation were also discovered by the model (sentences 2, 3, and 6). This implies that some important relations tend to be described in an equation. This result also indicates that the relations in which we are interested are significantly different from typical relations in other NLP tasks like ‘has_a’, ‘is_a’.

## 6. End-to-end system

**Figure 7.**
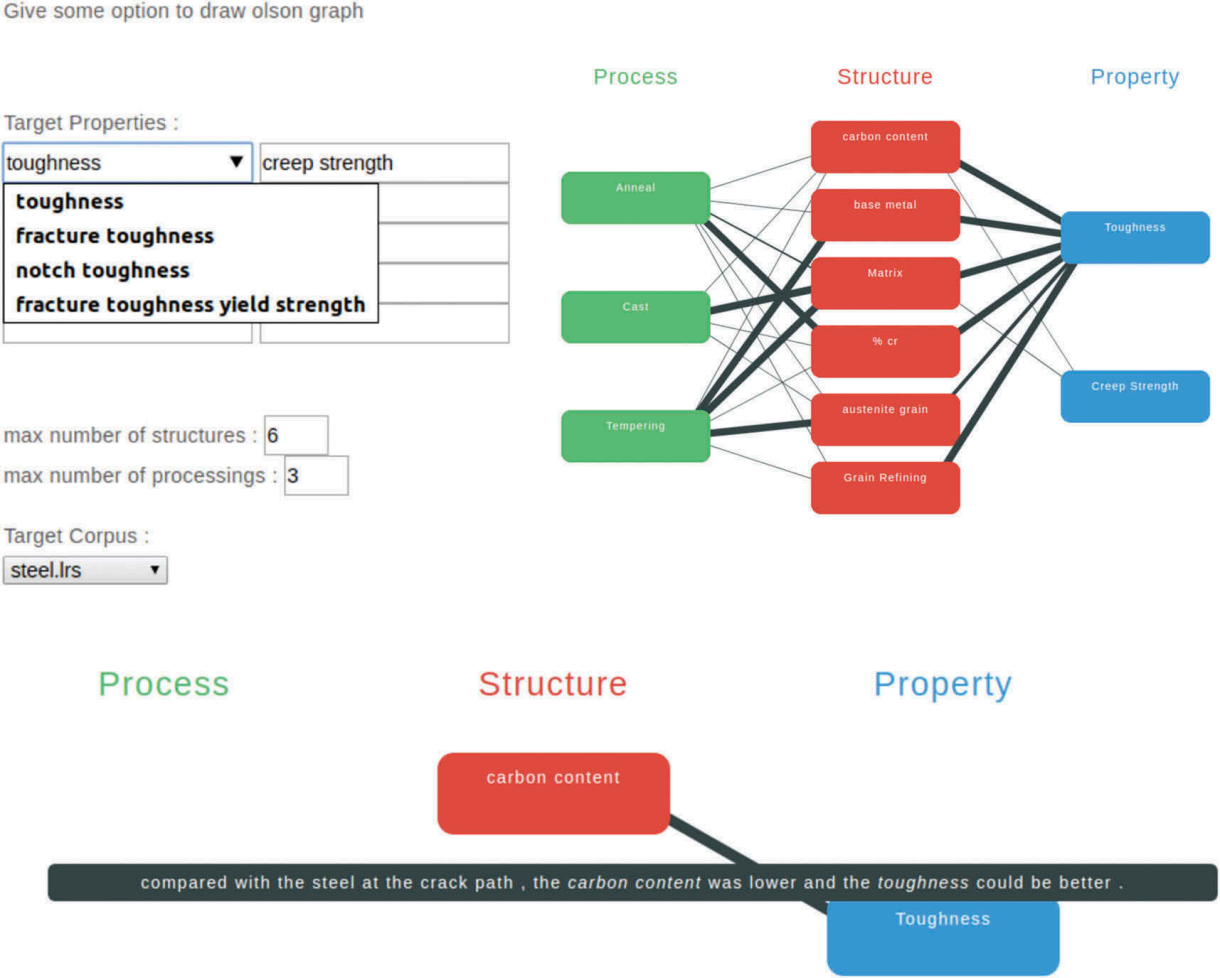
The end-to-end demo system. a) Desired properties and a base material were selected. b) A sample of the generated PSPP design chart. The desired properties were *toughness* and *creep strength*, and ‘steel’ was selected as base material. c) The representative sentence describing the relation between *toughness* and *carbon content.*

An end-to-end demo system was developed to test our CAMaD system on Apache Tomcat (http://tomcat.apache.org) as Figure 7. The demo system worked in a typical scenario, where a scientist was looking for factors related to certain desired properties. The demo system provides a PSPP design chart for the desired properties that the scientist provided.

The system input consisted of the desired properties along with a base material. The desired properties were selected from a list of properties collected as in Section 3.1. The base material was the target material, such as aluminum or titanium. It was important to obtain the desired knowledge. For example, the relationship between *strength* and *matrix* in titanium alloys might have been different from this relationship in aluminum alloys.

Thus, relationships were extracted from the scientific literature describing the base material. A set of studies was specified by the base material to find the desired knowledge. As in Section 4.2, the literature was collected by keyword search in ScienceDirect. All relations among the factors collected in Section 3.1 were scored by the CNN model as in Section 3.2 and some relationships were branched as described in Section 3.3.

The system output was a PSPP design chart suggesting the required structures and processes. The chart formed by three columns – process, structure, and property – suggested relations from the processes to the desired properties. Moreover, for each relation, a representative sentence for each factor pair was provided to justify the relation and aid the researcher’s understanding.

## 7. Conclusions and future work

In this study, we developed and tested a CAMaD system, a progressive knowledge extraction and representation system intended to support material design, by representing knowledge as relationships. Knowledge was represented as relationships in PSPP design charts. We leveraged weakly supervised learning for relation extraction. The end-to-end system proved our concept, and its relation identification performance was superior to that of other baseline models.

Further evaluation is the major feature of our work. In spite of the impressive results of relation extraction and brightness of the end-to-end system, a natural evaluation metric for the end-to-end task remains unclear. Additionally, system performances on other subtasks, factor collection and branching, are not evaluated in this study. Evaluations of these subtasks are not trivial. In factor collection, there are infinite number of factors in material design, and it is difficult to see the coverage of factors a system collected. In branching, there is no natural metric to compare or evaluate PSPP design charts. Thus, end-to-end evaluations are even more challenging.

We consider factor collection and mapping as the bottleneck of our system. The factor collection described in Section 3.1 and these factors were mapped into sentences that refer each factor in Section 3.2. Unlike in previous works [7,11], the factors were not named entities. Any noun phrase can be a factor, and factors were not predefined. At present, our system recognizes factors in a sentence using string matching. The obtained factors appear to be noisy and are not correctly categorized in some cases.

A natural extension of this task is multi-labeling. The relation we use at present is binary (pos/neg), and only identifies whether two factors are related. This label might be too abstract. In multi-labeling, a relation between factors is described with a label such as *produce, depress*, and *independent*. We believe multi-labeling renders the task more natural and informative.
